# Associations of Family Demands and Work–Life Conflict with Musculoskeletal Disorders among Korean Workers

**DOI:** 10.3390/ijerph15071419

**Published:** 2018-07-05

**Authors:** Young-Mee Kim, Sung-il Cho

**Affiliations:** Department of Public Health Science, Graduate School of Public Health, and Institute of Health and Environment, Seoul National University, 1 Gwanak-ro, Gwanak-gu, Seoul 08826, Korea; hihi415@snu.ac.kr

**Keywords:** family demands, work–life conflict, work–family conflict, musculoskeletal disorder, gender differences

## Abstract

Although family-related demands play a role in the effect of psychosocial work characteristics on health, research on work-related health has neglected the family domain. The aim of the present study was to identify the effects of family demands and work–life conflict (WLC) on musculoskeletal disorders (MSDs) among Korean workers. We analyzed data from the nationally representative Korean Working Conditions Survey conducted with 50,007 workers in 2014. Logistic regression analyses stratified by gender were performed to identify gender differences, and interaction terms including WLCs and key covariates were also incorporated. Childcare demands (odds ratio (OR), 1.16) were related to MSD only in male workers, whereas homemaking (OR, 1.09) and eldercare (OR, 1.26) demands were related to MSDs only in female workers. WLC was also associated with MSDs among both male (OR, 1.50) and female (OR, 1.55) workers. We found no gender difference in the effect of WLC on MSDs (*p* = 0.91). Moreover, childcare demands may exacerbate the effect of WLC on MSDs. Our data suggest that family demands and WLC could be important targets of workplace interventions to prevent MSDs, and future research should evaluate the role of family demands and WLC as stressors in the workplace.

## 1. Introduction

Musculoskeletal disorders (MSDs) are among the most common and costly health problems affecting working populations and constitute a major cause of disability [1]. Globally, the prevalence and burden from MSDs are exceptionally high [2]. It has been estimated that approximately 25–30% of European employees experience MSDs [3]. Indeed, 54.5% of adults in the United States, which amounts to 125 million people, have suffered from one or more MSD [4]. Moreover, MSDs cost the European Union (EU) 0.5–2% of gross domestic product (GDP) annually [5]. In the United States, the annual average direct cost of MSDs was estimated to be $576 billion, or 4.5% of GDP [6]. MSDs accounted for 21.3% of the years lived with disability (YLDs), behind only mental and behavioral problems (23.2%) [7].

Work-related causes of MSDs have been well established [8,9]. There is clear evidence that MSDs are directly caused by physical work conditions, such as heavy lifting and carrying, awkward postures, tiring positions, vibrations, and excessive repetitive motions [10]. Recently, a growing body of evidence has suggested that psychosocial work-related factors, such as high psychosocial job demands, low job control, low social support at work, and job stress, are also predictors or risk factors for MSD [11]. More recently, studies have focused on psychosocial aspects outside of the work environment, such as the impact of family and personal life, due to an increase in the number of women who work and changes in personal values [12,13,14]. However, little is known concerning associations between family demands, conflicts between work and home life, and MSDs or the possible moderating role of work demands in these relationships.

Work–family conflict (WFC) is a critical cause of mental and physical health problems [15,16]. WFC has been defined as “a form of inter-role conflict in which the role pressures from demands of work and family are incompatible, so that they negatively affect each other” [17]. WFC has become more important due to the increase in female education and labor-market participation as well as the rise in demand for eldercare due to aging of the population. Personal electronic communication devices, such as laptops and smart phones, have also increased the incidence of WFC [18]. WFC influences employee health outcomes and workplace productivity and is reportedly related to psychological stress, life and job satisfaction, substance abuse, and physical health [16,19,20,21]. Recent studies have used the term work–life conflict (WLC) to expand the scope of WFC to reflect nonwork demands and responsibilities beyond those related to one’s family [12,22].

Family demands are a key factor in WLC research [23,24]. “Family demands” refer to “the time spent, level of commitment to, and responsibilities related to family-related demands and obligations, such as housekeeping and caregiving for family members” [25]. Although family demands and WLC are key variables in investigations of the effects of the combination of work and family life on health, several studies did not evaluate important family demands other than WFC and working conditions [26]. Indeed, most focused on the relationship between WFC and health, but little about family demands, particularly in relation to domestic responsibilities, is known.

The relationships between family demands and health have been investigated. In Spain [27,28,29], demands in the family domain were related to self-perceived health status and health behaviors among females workers with a low educational level [27]. Regarding gender differences, family demands were not associated with the health of male workers but were associated with the psychosomatic symptoms and self-perceived health status of married female workers in family units consisting of more than three members [28]. In these studies, household size and living with an elderly relative or a child were used to assess family demands. Evidently, family demands depend not only on household size, having a child, and living with an elderly relative but also the degree of involvement in family-related work. However, these previous studies did not gather information on such factors [27,28,29]. A study of 239 Chinese employees reported that both work and family demands were related to employee stress, but family demands had the greater impact [25]. However, fewer male than female workers participated in the studies. The gender differences in the association between family demands and health suggest that this skewed gender distribution biased the results. Marchand et al. (2016) found that depressive symptoms were associated with WFC and couple-related problems but not with domestic tasks or caregiving among 1935 employees in 63 workplaces in Canada [30]. These studies did not consider several important confounders but did account for job stress and social engagements [25,30]. Furthermore, most of these studies were limited to white-collar workers, and skilled/unskilled blue-collar workers were excluded.

Family demands and WLC differ according to social and cultural context [21]. For instance, the average Korean worked for 2024 h per year in 2017, which places it third among Organization of Economic Cooperation and Development (OECD) countries (mean, 1759 h) [31]. That is, Koreans worked 20% more hours per year than residents of other OECD countries. Regarding the Global Gender Gap Index in 2017, Korea is ranked 118th of 144 countries [32]. Males spend 45 min per day on unpaid childcare and housework, whereas the comparable figure for females is 227.3 min [12]. Therefore, the effects of family demands and WLC on health by gender, especially in combination with other work demands, should be evaluated.

This study investigated whether family demands and WLC are related to MSDs among Korean workers by investigating three research questions. (1) Do family demands and WLC affect MSDs? (2) Are there gender differences in the relationships among family demands, WLC, and MSDs? (3) Do work and family demands exacerbate the effect of WLC on MSDs? Studies investigating the psychosocial work environments have mostly examined MSDs as the outcome variable. Abundant studies on the relationship between the psychosocial working environment and MSDs have been performed [33,34]. The current study differs from the existing literature by including family demands and WLC as psychosocial factors in the work environment and presents interesting empirical data according to gender. Our results highlight important psychosocial risk factors in the work environment. Also, prior research on family demands and WLC was limited to white-collar workers. We used a nationally representative survey dataset collected in 2014 that includes various subpopulations and occupations. We also evaluated the influence of various work- and life-related potential confounders, which have not been considered before (e.g., working hours, company size, physical demands at work, job stress, and social engagements). Moreover, the sample size of this study was large enough to examine interactions between variables that modify the effects of WLC in detail. Our results provide greater insight into population-based prevention strategies.

## 2. Materials and Methods

### 2.1. Data and Study Sample

We used data obtained from a representative sample of the working population of Korea by the fourth Korean Working Condition Survey (KWCS), which was conducted in 2014. The KWCS largely follows the European Working Conditions Survey (EWCS). The subjects of this study were workers at least 15 years of age who had performed paid work for more than 1 h during the previous week and lived in Korea. The response rate was 0.330, the cooperation rate was 0.699, and the refusal rate was 0.142 in the 2014 KWCS [35]. The validity and reliability of the 2014 KWCS, which are identical to those of the 2011 KWCS, have been confirmed [36]. We analyzed a total of 50,007 Korean workers: 25,247 males and 24,760 females.

### 2.2. Measures

#### 2.2.1. Family Demands

Subjects were asked about the frequency with which they undertook three household chores: “In general, how often are you involved in any of the following activities outside work (1) childcare demands, caring for and educating your children and/or grandchildren; (2) homemaking demands, cooking and housework; and (3) eldercare demands, caring for elderly/disabled relatives?” The possible responses were as follows: “1 h or more every day”, “every other day for less than 1 h”, “once or twice a week”, “once or twice a month”, “once or twice a year”, “never”, and “not applicable.” Subjects who responded “1 h or more every day” were defined as having family demands. 

#### 2.2.2. Work–Life Conflict

WLC was measured by three questions. (1) Work–life fit: “How well do your working hours fit in with your family or social commitments?” The possible responses to this question were: “very well”, “well”, “not very well”, and “not at all well”. Responses of “very well” or “well” were considered to indicate a good work–life fit, and responses of “not very well” or “not at all well” were considered to indicate a poor work–life fit. (2) The frequency of overtime work was evaluated with the following question: “Over the last 12 months, how often have you worked during your free time in order to meet work demands?” The possible responses were as follows: “nearly every day”, “once or twice a week”, “once or twice a month”, “less often”, and “never”. Responses of “nearly every day” or “once or twice a week” were considered to indicate a high frequency of overtime, and responses of “once or twice a month”, “less often”, or “never” were consider to reflect a low frequency of overtime. (3) Work-schedule adjustment was evaluated by the following question: “Which of the following describes your experience of arranging to take an hour or two off during working hours to take care of personal or family matters?” The possible responses to this question were as follows: “not difficult at all”, “not too difficult”, “somewhat difficult”, and “very difficult”. Responses of “not difficult at all” or “not too difficult” were considered indicative good work schedule adjustment, and responses of “somewhat difficult”, or “very difficult” were considered indicative of poor work-schedule adjustment. WLC was defined as a binary variable, being equal to 1 if answers to at least two of the above three questions (i.e., pertaining to poor work-life balance, frequent overtime, and/or poor work-schedule adjustment) indicated the existence of WLC. 

#### 2.2.3. Musculoskeletal Disorders

The presence of an MSD in the past 12 months was assessed with the following question: “Over the past 12 months, have you had any of the following health problems: backache, muscular pains in your shoulders, neck, and/or upper limbs; muscular pains in your lower limbs, such as your hips, legs, knees, feet, etc.?” The possible responses were “yes” and “no.”

#### 2.2.4. Confounding Variables

The following sociodemographic characteristics were evaluated: (15–39, 40–49, 50–59, or ≥60 years), education (less than middle school, high school, or college or above), monthly salary (<$1000, $1000–$1999, $2000–$2999, or ≥$3000), and employment type (self-employed, employee, or other). 

We selected work-related variables reportedly associated with family demands and MSDs [12,13,20]. Subjects were classified into five occupational groups: (1) managerial and professional (professional technicians or senior management); (2) white-collar; (3) sales and service; (4) skilled blue collar (skilled or semiskilled); and (5) unskilled and other (nonskilled, agriculture, or forestry). Subjects were classified as having nonshift or shift schedules, and company size was classified as <10, 10–99, or ≥100 employees. Working hours were divided into less or more than 48 h per week; the latter is considered overtime by the EWCS [37]. Job stress was assessed using the responses to the following statement: “I am under stress at work.” The possible responses were as follows: high job stress (always or most of the time) and low job stress (sometimes, not much, or not at all). Job-related physical demands were assessed with the following questions “Does your main paid job involve (1) tiring or painful positions; (2) lifting or moving people; (3) carrying or moving heavy loads; (4) standing; or (5) repetitive hand or arm movements.” Subjects responded on a seven-point scale ranging from 1 (never) to 7 (all the time). The sum of the item scores was used as the scale score, and these scores were dichotomized around the medians for the logistic regression analysis.

Subjects were asked about the frequency with which they participated in three types of social engagement: “In general, how often are you involved in any of the following activities outside work? (1) voluntary or charitable activity; (2) taking a training or educational course; and (3) sporting, cultural, or leisure activities.” The possible responses were as follows: “1 h or more daily”, “every other day for less than 1 h”, “once or twice a week”, “once or twice a month”, “once or twice a year”, “never”, and “not applicable”. Subjects who responded “more than once or twice a month” to either question (1) or (2) were considered to participate in volunteering or self-improvement activities, and those who responded “more than every other day for less than 1 h” to question (3) were considered to participate in leisure activities.

### 2.3. Statistical Analysis

Data from male and female workers were analyzed separately in this study. First, the chi-squared test was used to test the associations between independent variables and MSDs. Second, a multivariate logistic regression analysis was performed to examine the associations among family demands, WFC, and MSDs. We controlled for sociodemographic variables, potential work-related variables, which are proven protective factors for MSDs [38], and social engagements. The results are presented as odds ratios (ORs) with 95% confidence intervals. To identify gender differences in the effects of family demands and WLC on MSDs, we computed interaction terms between gender and WLC or family demands in a multiple logistic regression model (*n* = 50,007). Finally, to determine whether work and family demands exacerbated the effects of WLC on MSDs, we added the following interaction terms for WLC and work and family demands to the multiple logistic regression: employment type, working hours, physical demands at work, job stress, family demands, and social engagements. All analyses were performed using R software ver. 3.3 (R Foundation for Statistical Computing, Vienna, Austria).

## 3. Results

### 3.1. Sociodemographic Characteristics of the Study Subjects

The prevalence rates of self-reported MSDs during the previous year according to sociodemographic characteristics, work demands, family demands, and WLC are shown in Table 1. Respondents with missing information for educational level, salary, physical demands, or working hours were defined as the “no-response group.” Of the subjects, 50.5% were males and 49.5% were females. Of the female and male subjects, 29.0% and 26.8%, respectively, were 15–39 years old. The majority of subjects had a high school (38.5% of males and 40.9% of females) or college/university (43.1% of males and 34.0% of females) education. More than half the females (59.5%) had a monthly salary of less than $2000, whereas 63.0% of males had a monthly salary of more than $2000.

Regarding occupational category, almost half the males (44.8%) were skilled or unskilled blue-collar workers, whereas almost half the females (46.0%) were employed in sales and service. The majority of subjects (57.3% of males and 67.9% of females) were employed by a small company. Relatively few subjects did shift-work (8.8% of males and 5.3% of females). Of the male and female subjects, 40.2% and 33.9%, respectively, worked at least 48 h per week. Of these subjects, 22.5% of males and 21.5% of females suffered from job stress.

Regarding family demands, females consistently provided most of the family care, cleaning, cooking, and laundry; few males regularly engaged in these activities. Of the female subjects, 19.5%, 74.8%, and 2.2% reported childcare, homemaking, and eldercare demands, respectively; the proportions among males were considerably lower. However, 27.9% of males and 24.6% of females reported WLC. Regarding life-related activities, 3.7% of males and 4.6% of females regularly volunteered during their free time, and 16.7% of males and 16.0% of females attended a training or education course. Also, 12.9% of males and 10.8% of females participated in sporting, cultural, or leisure activities outside their home.

### 3.2. Prevalence of MSD by Work-Related Variables, Family Demand, and Social Engagements

Of the male and female subjects, 42.6% and 53.0%, respectively, reported having an MSD in the past 12 months, as shown in Table 1. Using aged under 30 years as a reference, we found that the prevalence of MSD increased with age up to 65 years. Of the male and female subjects, 66.7% and 78.0%, respectively, of those with less than a secondary education reported having an MSD, which was higher than those with a high school or university education. Subjects with low salaries reported a higher prevalence of MSDs. Regarding occupational category, male (57.0% and 53.1% for skilled blue-collar workers and unskilled workers and others, respectively) and female (75.9% and 68.9% for skilled blue-collar workers and for unskilled workers and others, respectively) subjects had a higher prevalence of MSDs than did white-collar or sales and service workers. Male (49.0%) and female (59.1%) subjects who worked more than 48 h per week had a higher prevalence of MSDs than those who worked less than 48 h per week. Subjects employed in physically demanding occupations (56.1% of males and 66.3% of females) had a higher prevalence of MSDs than those in nonphysically demanding occupations.

Regarding family demands, subjects of both genders without childcare demands (43.1% of males and 55.7% of females) reported a higher prevalence of MSDs than those with childcare demands. Female workers with high homemaking demands and eldercare had a higher prevalence of MSDs than those without such demands (55.5% for homemaking demands and 67.6% for eldercare demands). Regarding social involvement, subjects of both genders who volunteered or engaged in self-development or leisure activities had a lower prevalence of MSDs than did subjects without such engagements. Male (48.9%) and female (59.3%) subjects who reported experiencing WLC had a higher prevalence of MSDs than did those without WLC.

### 3.3. Prevalence of Family Demands by Key Covariates

As shown in Table 2, females consistently performed most of the family care, cleaning, cooking, and laundry; few males regularly engaged in these activities. Of the female subjects, 19.5% were engaged in childcare for more than 1 h a day, and 2.2% were engaged in eldercare for more than 1 h a day. The corresponding values in males were 5.9% and 0.7%, respectively. Subjects of both genders under 50 years of age reported higher childcare demands, whereas those over 60 years of age reported higher eldercare demands. Regarding housework demands, 74.8% of females were engaged in housework for over 1 h a day, which was around sixfold higher than the comparable figure for males. Among females, the majority of skilled (82.9%) and unskilled blue-collar workers (84.5%) had higher housework demands than white-collar workers (64.5%) or sales and service workers (74.4%). Subjects with a highly physically demanding job reported higher homemaking demands than those without such a job; this was the case for both males (14.1%) and females (77.5%). Among males, subjects who volunteered or participated in self-development activities had higher childcare and homemaking demands. The prevalence of family demands did not differ according to work hours.

### 3.4. Relationships among Family Demands, WLC, and MSDs

Regarding working conditions, being employed (OR, 0.74; 95% CI, 0.68–0.81 for males; OR, 0.88; 95% CI, 0.80–0.95 for females), being a skilled worker (OR, 1.51; 95% CI, 1.37–1.67 for males; OR, 1.77; 95% CI, 1.55–2.03 for females), being an unskilled worker (OR, 1.33; 95% CI, 1.17–1.50 for males; OR, 1.44; 95% CI, 1.25–1.66 for females), work hours (OR, 1.28; 95% CI, 1.20–1.36 for males; OR, 1.21; 95% CI, 1.13–1.30 for females), and a physically demanding job (OR, 2.12; 95% CI, 2.00–2.24 for males; OR, 2.26; 95% CI, 2.13–2.40 for females) were associated with MSDs. However, job stress (OR, 1.11; 95% CI, 1.04–1.18) and participation in leisure activities (OR, 0.77; 95% CI, 0.71–0.84) were related to MSDs only among male workers. Shift type (OR, 1.17; 95% CI, 1.03–1.32) was related to MSDs only among female workers.

After adjusting for work-related variables and key covariates, the effect of family demands on the prevalence of MSDs differed by gender, as shown in Table 3. The effects of family demands on MSD differed by gender. Childcare demands (OR, 1.16; 95% CI, 1.03–1.31) were related to MSDs only in male workers, while homemaking demands (OR, 1.09; 95% CI, 1.02–1.17) and eldercare demands (OR, 1.26; 95% CI, 1.03–1.55) were related to MSDs only in female workers. WLC was significantly associated with MSDs among male (OR, 1.50; 95% CI, 1.41–1.60) and female (OR, 1.55; 95% CI, 1.45–1.65) workers. The effect of WLC on MSDs did not differ by gender (*p* = 0.91).

### 3.5. Work and Family Demands Exert a Moderating Effect on the Relationship between WLC and MSDs

Significant modifying effects of WLC and certain work and family demands on MSDs were found, as shown in Table 4. Although childcare demands (OR, 1.00; 95% CI, 0.93–1.08) were not associated with MSDs in females, the interaction of WLC with childcare demands was significant in both males (OR, 2.26) and females (OR, 1.69). However, the interactions of WLC with job stress (OR, 1.75) and a physically demanding job (OR, 3.60) were significant only for females.

## 4. Discussion

We investigated the effects of family demands and WLC on MSDs using nationally representative data collected in 2014. WLC was related to MSDs among male and female workers after adjusting for work-related factors and social engagements. In addition, our findings show that family demands are associated with MSD prevalence, which, to the best of our knowledge, has never been reported previously.

The main contributions of this study can be summarized as follows. First, for the first time, we interpreted family demands and WLC as psychosocial factors in working environments and showed that they had significant relationships with MSDs. This result indicates that researchers should treat family demands and WLC as risk factors when examining the association between the psychosocial working environment and health problems. Second, ours is the first study to investigate whether there were gender differences in the relationships among family demands, WLC, and MSDs. Third, we found that childcare demands significantly modified the relationship between WLC and MSDs. The identification of effect modifiers implies that these factors contribute to the causal mechanisms of health hazards arising from WLC.

Family demands and WFC can evoke physiological stress reactions. A disparity between the demands placed on and the abilities of workers results in negative emotional and physiological responses [39]. For example, although employees who work long hours might expect to do less housework, this may not actually be the case [40], which increases their negative psychological reaction to family demands [25]. Also, employees who experience WFC may be preoccupied with family-related demands, such as caregiving [20]. The psychological distress generated by a situation perceived to be uncontrollable can cause persistent feelings of distress and subsequently influence health [39]. Psychological distress can increase muscular tension, resulting in MSDs [41,42]. Psychosocial factors can also influence mechanical load by promoting unfavorable body postures [43]. Indeed, psychological stressors reportedly increase trapezius muscle activity [44,45]. These results support the existence of a biomechanical pathway between work-related psychological factors and MSDs.

The prevalence of WLC was similar between male and female workers in this study, but females tended to respond to family demands more frequently than males in this study. The roles and responsibilities of males and females within the family have become more egalitarian with increasing female workforce participation [46]. For example, nationally representative data from the United States indicated that attitudes toward male and female work and family roles became more equitable between the 1970s and the 2010s [47]. Therefore, male workers are more likely to experience WFC than before. However, males tend to be less effective than females in such roles [48]. Employed females still feel more responsible for the family and tend to engage in household tasks and caregiving more frequently than do males [49].

The effect of WLC on the prevalence of MSDs did not differ significantly according to gender in our study, which is consistent with previous reports [12,14]. Gender differences in health outcomes, such as stress, anxiety, and depression, have been previously reported [50,51]. However, no gender difference in the associations between WLC and health outcomes (e.g., anxiety, stress, lack of sleep, poor self-assessed health, poor physical health, and health behaviors) was noted [52,53,54,55]. WLC is significantly related to MSDs in male and female workers [12,14]. 

We found that the types of family demands affecting health differed by gender; namely, men were more affected by childcare responsibilities, whereas women were more affected by homemaking and eldercare. The associations between family demands and health problems—that is, life stress, self-perceived health status, chronic conditions, psychosocial symptoms, and depression—reportedly differ by gender [28,29,30]. Among male workers, family demands were not related to health-related outcomes. However, family demands were associated with poor self-perceived health status and psychosocial symptoms among married female workers who lived with more than three members [28]. Caregiving can exert positive and negative effects on health [56]. Caring for a family member was related to psychosocial stress but also offered benefits, such as closeness with that family member. 

The health effects of WLC showed a significant interaction with childcare but not with other family demands. The association between WLC and MSDs was stronger among male and female workers who had childcare responsibilities. Childcare responsibilities may afford less control over the schedule and quantity of work compared with homemaking demands. Eldercare responsibilities may be similar in this regard, but the frequency of eldercare was very low among the working population in this study, possibly resulting in insufficient power to detect a significant interaction. These results suggest that social policies supporting childcare activities are important to relieve WLC and its associated health risks in male and female workers.

Policies to address WLC have been implemented in Korea as well as in other countries. The foundational legislation of work-family policies in Korea is contained within the “Act on Equal Employment and Support for Work-Family Reconciliation”. This Act was reformed in 2007, cementing its importance to work-family balance. Another law, the “Act on the Promotion of Creation of Family-Friendly Social Environment” was also enacted in 2007 to promote family-friendly communities. As of 2014, when our survey in was conducted, the prevalence of WFC still remained relatively high. The aforementioned laws are considered insufficient to meet the needs of a working family [57]. A recent evaluation study called for the revision of these laws to overcome their limitations [58]. 

This study had several methodological limitations. First, it used cross-sectional data, which prevented determination of causality; therefore, reverse or reciprocal causation cannot be excluded. Second, family demands, WLC, and MSDs were assessed with self-reported questionnaires, which can be influenced by subjective experience. Moreover, the presence of MSDs was assessed using a single item, and the number of musculoskeletal pain sites was not considered. This may have resulted in an underestimation of the strength of the relationships among family demands, WLC, and MSDs. Third, we addressed missing values using the missing indicator method [59]. In this study, 0.8%, 6.9%, 1.1%, and 1.6% of subjects had missing information concerning educational level, salaries, working hours, and physical demands, respectively. The logistic regression results were comparable to those of a complete case analysis after omitting subjects with missing data (*n* = 45,001).

## 5. Conclusions

Our study provides new empirical evidence that the risks of MSDs among workers are associated with family demands and WLC, independent of work demands. More specifically, high childcare demands may increase the adverse effect of WLC on MSDs in both men and women. In our study population, gender differences were not found in the effect of WLC on MSDs. However, women experienced more adverse health effects of family demands than men. According to our findings, prevention of MSDs in workers requires appropriate coordination of family demands as well as a reduction of WLC, both for male and female workers. 

## Figures and Tables

**Table 1:** Demographic characteristics of the study population and prevalence of MSDs in the past 12 months *n* (%).

Characteristic		Males (*n* = 25,247)				Females (*n* = 24,760)		
		Without MSD *n* = 14,499 (57.4)	With MSD *n* = 10,748 (42.6)	*p* ^a^		Without MSD *n* = 11,632 (47.0)	With MSD *n* = 13,128 (53.0)	*p* ^a^
Age (years)								
15–39	7332 (29.0)	5301 (72.3)	2031 (27.7)	<0.01	6646 (26.8)	4363 (65.6)	2283 (34.4)	<0.01
40–49	6424 (25.4)	3878 (60.4)	2546 (39.6)		6856 (27.7)	3608 (52.6)	3248 (47.4)	
50–59	5932 (23.5)	3150 (53.1)	2782 (46.9)		6028 (24.3)	2528 (41.9)	3500 (58.1)	
≥60	5559 (22.0)	2170 (39.0)	3389 (61.0)		5230 (21.2)	1133 (21.7)	4097 (78.3)	
Educational level								
Below secondary	4449 (17.6)	1483 (33.3)	2966 (66.7)	<0.01	6011 (24.3)	1322 (22.0)	4689 (78.0)	<0.01
High school	9710 (38.5)	5335 (54.9)	4375 (45.1)		10,136 (40.9)	4954 (48.9)	5182 (51.1)	
College or above	10,879 (43.1)	7564 (69.5)	3315 (30.5)		8414 (34.0)	5279 (62.7)	3135 (37.3)	
No response	209 (0.8)	117 (56.0)	92 (44.0)		199 (0.8)	77 (38.7)	122 (61.3)	
Employment type								
Self-employed	9390 (37.2)	4414 (47.0)	4976 (53.0)	<0.01	7436 (30.0)	2836 (38.1)	4600 (61.9)	<0.01
Employee	15,742 (62.4)	10,015 (63.6)	5727 (36.4)		15,009 (60.6)	8047 (53.6)	6962 (46.4)	
Employer	115 (0.4)	70 (60.9)	45 (39.1)		2315 (9.3)	749 (32.4)	1566 (67.6)	
Salary (USD * per month)								
<1000	2682 (10.6)	1197 (44.6)	1485 (55.4)	<0.01	5269 (21.3)	1872 (35.5)	3397 (64.5)	<0.01
1000–1999	5897 (23.4)	3049 (51.7)	2848 (48.3)		9454 (38.2)	4740 (50.1)	4714 (49.9)	
2000–2999	7306 (28.9)	4285 (58.7)	3021 (41.3)		4654 (18.8)	2599 (55.8)	2055 (44.2)	
>2999	8598 (34.1)	5533 (64.4)	3065 (35.6)		2656 (10.7)	1436 (54.1)	1220 (45.9)	
No response	764 (3.0)	435 (56.9)	329 (43.1)		2727 (11.0)	958 (36.1)	1742 (63.9)	
Occupational category								
Managerial and professional	3502 (13.9)	2401 (68.6)	1101 (31.4)	<0.01	2703 (10.9)	1714 (63.4)	989 (36.6)	<0.01
White collar	4170 (16.5)	3143 (75.4)	1027 (24.6)		3884 (15.7)	2623 (67.5)	1261 (32.5)	
Sales and service	6247 (24.7)	3968 (63.5)	2279 (36.5)		11,397 (46.0)	5468 (48.0)	5929 (52.0)	
Skilled blue collar	8388 (33.2)	3608 (43.0)	4780 (57.0)		4018 (16.2)	969 (24.1)	3049 (75.9)	
Unskilled and other	2940 (11.6)	1379 (46.9)	1561 (53.1)		2758 (11.1)	858 (31.1)	1900 (68.9)	
Company size (number of people)								
1–9	14,470 (57.3)	7431 (51.4)	7039 (48.6)	<0.01	16,814 (67.9)	7184 (42.7)	9630 (57.3)	<0.01
10–99	6873 (27.2)	4466 (65.0)	2407 (35.0)		6028 (24.3)	3366 (55.8)	2662 (44.2)	
≥100	3904 (15.5)	2602 (66.6)	1302 (33.4)		1918 (7.7)	1082 (56.4)	836 (43.6)	
Shift type								
Nonshift	23,034 (91.2)	13,204 (57.3)	9830 (42.7)	0.28	23,437 (94.7)	11,015 (47.0)	12,422 (53.0)	0.79
Shift	2213 (8.8)	1295 (58.5)	918 (41.5)		1323 (5.3)	617 (46.6)	706 (53.4)	
Work hours per week								
<48	14,792 (58.6)	9192 (62.1)	5600 (37.9)	<0.01	16,090 (65.0)	8098 (50.3)	7992 (49.7)	<0.01
≥48	10,145 (40.2)	5174 (51.0)	4971 (49.0)		8398 (33.9)	3433 (40.9)	4965 (59.1)	
No response	310 (1.2)	133 (42.9)	177 (57.1)		272 (1.1)	101 (37.1)	171 (62.9)	
Job stress								
No	19,569 (77.5)	11,248 (57.5)	8321 (42.5)	0.76	19,429 (78.5)	9032 (46.5)	10,397 (53.5)	<0.01
Yes	5678 (22.5)	3251 (57.3)	2427 (42.7)		5331 (21.5)	2600 (48.8)	2731 (51.2)	
Physical demands								
Low	13,516 (53.5)	9283 (68.7)	4233 (31.3)	<0.01	12,683 (51.2)	7507 (59.2)	5176 (40.8)	<0.01
High	11,301 (44.8)	4963 (43.9)	6338 (56.1)		11,687 (47.2)	3934 (33.7)	7753 (66.3)	
No response	430 (1.7)	253 (58.8)	177 (41.2)		390 (1.6)	191 (49.0)	199 (51.0)	
Family demands								
Childcare demands								
No	23,768 (94.1)	13,528 (56.9)	10,240 (43.1)	<0.01	19,944 (80.5)	8838 (44.3)	11,106 (55.7)	<0.01
Yes	1479 (5.9)	971 (65.7)	508 (34.3)		4816 (19.5)	2794 (58.0)	2022 (42.0)	
Homemaking demands								
No	21,995 (87.1)	12,675 (57.6)	9320 (42.4)	0.10	6246 (25.2)	3392 (54.3)	2854 (45.7)	<0.01
Yes	3252 (12.9)	1824 (56.1)	1428 (43.9)		18,514 (74.8)	8240 (44.5)	10,274 (55.5)	
Eldercare demands								
No	25,074 (99.3)	14,402 (57.4)	10,672 (42.6)	0.72	24,223 (97.8)	11,458 (47.3)	12,765 (52.7)	<0.01
Yes	173 (0.7)	97 (56.1)	76 (43.9)		537 (2.2)	174 (32.4)	363 (67.6)	
Social involvement								
Volunteering								
No	24,318 (96.3)	13,892 (57.1)	10,426 (42.9)	<0.01	23,627 (95.4)	10,988 (46.5)	12,639 (53.5)	<0.01
Yes	929 (3.7)	607 (65.3)	322 (34.7)		1133 (4.6)	644 (56.8)	489 (43.2)	
Self-development								
No	21,032 (83.3)	11,599 (55.1)	9433 (44.9)	<0.01	20,797 (84.0)	9292 (44.7)	11,505 (55.3)	<0.01
Yes	4215 (16.7)	2900 (68.8)	1315 (31.2)		3963 (16.0)	2340 (59.0)	1623 (41.0)	
Leisure activities								
No	21,985 (87.1)	12,320 (56.0)	9665 (44.0)	<0.01	22,081 (89.2)	10,224 (46.3)	11,857 (53.7)	<0.01
Yes	3262 (12.9)	2179 (66.8)	1083 (33.2)		2679 (10.8)	1408 (52.6)	1271 (47.4)	
Work–life conflict								
No	18,192 (72.1)	10,891 (59.9)	7301 (40.1)	<0.01	18,680 (75.4)	9160 (49.0)	9520 (51.0)	<0.01
Yes	7055 (27.9)	3608 (51.1)	3477 (48.9)		6080 (24.6)	2472 (40.7)	3608 (59.3)	

Percentages in the shaded areas are the column percentages of each variable. Other percentages are row percentages by gender. * In the survey year, the exchange rate was 1000 KRW = 0.94 USD. ^a^ Chi-squared test. In terms of employment status, 37.2% of males and 30.0% of females were self-employed.

**Table 2:** Prevalence of family demands by sociodemographic variables, work demands, and WLC, *n* (%).

Characteristic	Males (*n* = 25,247)			Females (*n* = 24,760)		
	Childcare *n* = 1479 (5.9)	Homemaking *n* = 3252 (12.9)	Eldercare *n* = 173 (0.7)	Childcare *n* = 4816 (19.5)	Homemaking *n* = 18,514 (74.8)	Eldercare *n* = 537 (2.2)
Age						
15–39 years	817 (11.1)	1096 (14.9)	27 (0.4)	2037 (30.7)	3641 (54.8)	53 (0.8)
40–49 years	545 (8.5)	690 (10.7)	52 (0.8)	2313 (33.7)	5579 (81.4)	117 (1.7)
50–59 years	99 (1.7)	650 (11.0)	45 (0.8)	370 (6.1)	4945 (82.0)	136 (2.3)
60 years and older	18 (0.3)	816 (14.7)	49 (0.9)	96 (1.8)	4349 (83.2)	231 (4.4)
Educational level						
Less than secondary education	29 (0.7)	818 (18.4)	47 (1.1)	133 (2.2)	5067 (84.3)	259 (4.3)
High school	404 (4.2)	1226 (12.6)	61 (0.6)	1907 (18.8)	7827 (77.2)	166 (1.6)
College or above	1043 (9.6)	1188 (10.9)	62 (0.6)	2754 (32.7)	5510 (65.5)	109 (1.3)
No answer	3 (1.4)	20 (9.6)	3 (1.4)	22 (11.1)	110 (55.3)	3 (1.5)
Salary (USD * per month)						
<1000	40 (1.5)	617 (23.0)	39 (1.5)	577 (11.0)	4263 (80.9)	228 (4.3)
1000–1999	184 (3.1)	988 (16.8)	59 (1.0)	2049 (21.7)	7061 (74.7)	170 (1.8)
2000–2999	538 (7.4)	880 (12.0)	34 (0.5)	1163 (25.0)	3154 (67.8)	51 (1.1)
>2999	680 (7.9)	670 (7.8)	35 (0.5)	635 (23.9)	1774 (66.8)	42 (1.6)
No answer	37 (4.8)	97 (12.7)	6 (0.8)	392 (14.4)	2262 (82.9)	46 (1.7)
Employment type						
Self-employed	333 (3.5)	1176 (12.5)	83 (0.9)	987 (13.2)	5785 (77.8)	174 (2.3)
Employee	1142 (7.3)	2059 (13.1)	90 (0.6)	3552 (23.7)	10,711 (71.4)	326 (2.2)
Employs others	4 (3.5)	17 (14.8)	0 (0.0)	286 (12.4)	2018 (87.2)	37 (1.6)
Occupational category						
Managerial and professional	335 (9.6)	374 (10.7)	23 (0.7)	985 (36.4)	1872 (69.3)	45 (1.7)
White collar	397 (9.5)	421 (10.1)	15 (0.4)	1274 (32.8)	2504 (64.5)	44 (1.1)
Sales and service	287 (4.6)	843 (13.5)	43 (0.7)	1980 (17.4)	8476 (74.4)	209 (1.8)
Skilled blue collar	385 (4.6)	1085 (12.9)	63 (0.8)	293 (7.3)	3331 (82.9)	126 (3.1)
Unskilled and other	75 (2.6)	529 (18.0)	29 (1.0)	284 (10.3)	2331 (84.5)	113 (4.1)
Company size						
1–9	593 (4.1)	1981 (13.7)	110 (0.8)	2769 (16.5)	12,795 (76.1)	347 (2.1)
10–99	501 (7.3)	848 (12.3)	42 (0.6)	1578 (26.2)	4393 (72.9)	147 (2.4)
>99	385 (9.9)	423 (10.8)	21 (0.5)	469 (24.5)	1326 (69.1)	43 (2.2)
Shift type						
Nonshift	1334 (5.8)	2975 (12.9)	156 (0.7)	4538 (19.4)	17,622 (75.2)	511 (2.2)
Shift	145 (6.6)	277 (12.5)	17 (0.8)	278 (21.0)	892 (67.4)	26 (2.0)
Work hours (per week)						
<48	981 (6.6)	2027 (13.7)	103 (0.7)	3546 (22.0)	12,109 (75.3)	403 (2.5)
≥48	484 (4.8)	1190 (11.7)	66 (0.7)	1225 (14.6)	6206 (73.9)	128 (1.5)
No answer	14 (4.5)	35 (11.3)	4 (1.3)	45 (16.5)	199 (73.2)	6 (2.2)
Job stress						
No	1144 (5.8)	2480 (12.7)	129 (0.7)	3662 (18.8)	14,646 (75.4)	423 (2.2)
Yes	335 (5.9)	772 (13.6)	44 (0.8)	1154 (21.6)	3868 (72.6)	114 (2.1)
Physical demands						
Low	911 (6.7)	1604 (11.9)	82 (0.6)	2868 (22.6)	9174 (72.3)	210 (1.7)
High	535 (4.7)	1596 (14.1)	86 (0.8)	1878 (16.1)	9057 (77.5)	321 (2.7)
No answer	33 (7.7)	52 (12.1)	5 (1.2)	70 (17.9)	283 (72.6)	6 (1.5)
Volunteer						
No	1400 (5.8)	3100 (12.7)	163 (0.7)	4536 (19.2)	17,629 (74.6)	485 (2.1)
Yes	79 (8.5)	152 (16.4)	10 (1.1)	280 (24.7)	885 (78.1)	52 (4.6)
Self-development						
No	1045 (5.0)	2636 (12.5)	134 (0.6)	3698 (17.8)	15,836 (76.1)	452 (2.2)
Yes	434 (10.3)	616 (14.6)	39 (0.9)	1118 (28.2)	2678 (67.6)	85 (2.1)
Leisure activity						
No	1249 (5.7)	2708 (12.3)	149 (0.7)	4279 (19.4)	16,523 (74.8)	481 (2.2)
Yes	230 (7.1)	544 (16.7)	24 (0.7)	537 (20.0)	1991 (74.3)	56 (2.1)
Work–life conflict						
No	1077 (5.9)	2404 (13.2)	148 (0.8)	3574 (19.1)	14,134 (75.7)	430 (2.3)
Yes	402 (5.7)	848 (12.0)	28 (0.4)	1242 (20.4)	4380 (72.0)	107 (1.8)

* In the survey year, the exchange rate was 1000 KRW = 0.94 USD.

**Table 3:** Associations among family demands, WLC, and MSDs in the past 12 months.

	Odds Ratio (95% Confidence Intervals)	
	Male Workers (*n* = 25,247)	Female Workers (*n* = 24,760)
Employment type		
Self-employed	1.00	1.00
Employee	0.74 (0.68–0.81) ^‡^	0.88 (0.80–0.95) ^‡^
Employs others	0.56 (0.37–0.85) ^†^	0.99 (0.81–1.20)
Salary (USD * per month)		
<1000	1.00	1.00
1000–1999	0.93 (0.83–1.03)	1.00 (0.92–1.09)
2000–2999	0.99 (0.89–1.11)	1.03 (0.93–1.14)
>2999	0.89 (0.79–1.01)	1.07 (0.95–1.21)
No answer	1.02 (0.84–1.23)	1.03 (0.85–1.24)
Occupational category		
Managerial and professional	1.00	1.00
White collar	0.98 (0.88–1.09)	1.09 (0.98–1.22)
Sales and service	0.83 (0.75–0.92) ^‡^	1.08 (0.97–1.20)
Skilled blue collar	1.51 (1.37–1.67) ^‡^	1.77 (1.55–2.03) ^‡^
Unskilled and other	1.33 (1.17–1.50) ^‡^	1.44 (1.25–1.66) ^‡^
Shift type		
Nonshift	1.00	1.00
Shift	0.94 (0.85–1.03)	1.17 (1.03–1.32) ^†^
Work hours (per week)		
<48	1.00	1.00
≥48	1.28 (1.20–1.36) ^‡^	1.21 (1.13–1.30) ^‡^
No answer	1.52 (1.19–1.94) ^‡^	1.30 (0.99–1.71)
Job stress		
No	1.00	1.00
Yes	1.11 (1.04–1.18) ^‡^	1.02 (0.95–1.09)
Physical demands		
Low	1.00	1.00
High	2.12 (2.00–2.24) ^‡^	2.26 (2.13–2.40) ^‡^
No answer	1.33 (1.08–1.64) ^‡^	1.40 (1.13–1.74) ^‡^
Family demands		
Childcare demands		
No	1.00	1.00
Yes	1.16 (1.03–1.31) ^†^	1.00 (0.93–1.08)
Homemaking demands		
No	1.00	1.00
Yes	0.99 (0.91–1.08)	1.09 (1.02–1.17) ^†^
Eldercare demands		
No	1.00	1.00
Yes	0.86 (0.62–1.19)	1.26 (1.03–1.55) ^†^
Social involvements		
Volunteer		
No	1.00	1.00
Yes	0.92 (0.79–1.07)	0.88 (0.77–1.01)
Self-development		
No	1.00	1.00
Yes	0.94 (0.87–1.02)	1.01 (0.93–1.09)
Leisure activity		
No	1.00	1.00
Yes	0.77 (0.71–0.84) ^‡^	1.03 (0.94–1.13)
Work–life conflicts		
No	1.00	1.00
Yes	1.50 (1.41–1.60) ^‡^	1.55 (1.45–1.65) ^‡^

^†^ *p* < 0.05, ^‡^ *p* < 0.01, Odds ratios were adjusted for age, education level, family type, company size, and all other variables in Table 1.

**Table 4:** Odds ratios for associations of MSDs and WLC with work and family demands with significant interaction effects.

		Males (*n* = 25,247)		Females (*n* = 24,760)	
		Work–Life Conflict			
		No	Yes	No	Yes
Model 1	Job stress				
	No	1	1.49 ^‡^	1	1.46 ^‡^
	Yes	1.09 ^†^	1.62	0.95	**1.75**
Model 2	Physical demands				
	Low	1	1.52 ^‡^	1	1.43 ^‡^
	High	2.13 ^‡^	3.14	2.19 ^‡^	**3.60**
	No answer	1.28 ^†^	2.22	1.27	2.80
Model 3	Childcare demands				
	Low	1	1.47 ^‡^	1	1.49 ^‡^
	High	1.02	**2.26**	0.96	**1.69**

^†^ *p* < 0.05, ^‡^ *p* < 0.01. An underlined OR for the combined effects indicates that the interaction term is significant (*p* < 0.05). Models were adjusted for age, educational level, family type, company size, and all other variables in the Table 1.
